# The effect of volumetric breast density on the risk of screen-detected and interval breast cancers: a cohort study

**DOI:** 10.1186/s13058-017-0859-9

**Published:** 2017-06-05

**Authors:** Johanna O. P. Wanders, Katharina Holland, Nico Karssemeijer, Petra H. M. Peeters, Wouter B. Veldhuis, Ritse M. Mann, Carla H. van Gils

**Affiliations:** 1 Julius Center for Health Sciences and Primary Care, University Medical Center Utrecht, P.O. Box 85500, 3508 GA Utrecht, The Netherlands; 2 Department of Radiology and Nuclear Medicine, Radboud University Medical Center, Geert Grooteplein 10, 6525 GA Nijmegen, The Netherlands; 3 MRC-PHE Centre for Environment and Health, Department of Epidemiology and Biostatistics, School of Public Health, Imperial College London, St. Mary’s Campus, Norfolk Place, W2 1PG London, UK; 4 Department of Radiology, University Medical Center Utrecht, P.O. Box 85500, 3508 GA Utrecht, The Netherlands

**Keywords:** Volumetric mammographic breast density, Mammography screening, Interval breast cancer risk

## Abstract

**Background:**

In the light of the breast density legislation in the USA, it is important to know a woman’s breast cancer risk, but particularly her risk of a tumor that is not detected through mammographic screening (interval cancer). Therefore, we examined the associations of automatically measured volumetric breast density with screen-detected and interval cancer risk, separately.

**Methods:**

Volumetric breast measures were assessed automatically using Volpara version 1.5.0 (Matakina, New Zealand) for the first available digital mammography (DM) examination of 52,814 women (age 50 − 75 years) participating in the Dutch biennial breast cancer screening program between 2003 and 2011. Breast cancer information was obtained from the screening registration system and through linkage with the Netherlands Cancer Registry. We excluded all screen-detected breast cancers diagnosed as a result of the first digital screening examination. During a median follow-up period of 4.2 (IQR 2.0–6.2) years, 523 women were diagnosed with breast cancer of which 299 were screen-detected and 224 were interval breast cancers. The associations between volumetric breast measures and breast cancer risk were determined using Cox proportional hazards analyses.

**Results:**

Percentage dense volume was found to be positively associated with both interval and screen-detected breast cancers (hazard ratio (HR) 8.37 (95% CI 4.34–16.17) and HR 1.39 (95% CI 0.82–2.36), respectively, for Volpara density grade category (VDG) 4 compared to VDG1 (*p* for heterogeneity < 0.001)). Dense volume (DV) was also found to be positively associated with both interval and screen-detected breast cancers (HR 4.92 (95% CI 2.98–8.12) and HR 2.30 (95% CI 1.39–3.80), respectively, for VDG-like category (C)4 compared to C1 (*p* for heterogeneity = 0.041)). The association between percentage dense volume categories and interval breast cancer risk (HR 8.37) was not significantly stronger than the association between absolute dense volume categories and interval breast cancer risk (HR 4.92).

**Conclusions:**

Our results suggest that both absolute dense volume and percentage dense volume are strong markers of breast cancer risk, but that they are even stronger markers for predicting the occurrence of tumors that are not detected during mammography breast cancer screening.

**Electronic supplementary material:**

The online version of this article (doi:10.1186/s13058-017-0859-9) contains supplementary material, which is available to authorized users.

## Background

The female breast consists of fibroglandular (dense) and fat (nondense) tissue. It is known that breast density, measured as either the percentage or absolute amount of fibroglandular tissue in the breast, is a strong breast cancer risk factor [1–3]. Besides this, high breast density is also known to hinder the detection of tumors during mammographic screening [4–6]. These findings have led to legislation in the USA that mandates disclosure of breast density information to women undergoing mammography screening. Depending on their breast density, women may opt for supplemental screening, for example by ultrasound or magnetic resonance imaging (MRI).

When breast density measures are used as a tool to stratify women for different screening strategies, it is important that these measures are highly reproducible as it is undesirable that the likelihood of a woman being classified as having dense breasts is dependent on which radiologist she goes to. Another prerequisite is that these measures can be obtained in the daily practice of high-throughput screening.

Breast density can be measured using several methods. The most widely used methods are visual estimation using Breast Imaging - Reporting and Data System (BI-RADS) density categories and the semi-automatic area-based Cumulus method [7]. The former is mostly used by radiologists in clinical and screening practice. The latter is mostly used for research purposes. Both methods have been shown to be related to breast cancer risk. However, they also have some disadvantages. BI-RADS density classification has high inter-reader variability and Cumulus is very time-consuming. Hence, neither method is optimal for stratification in breast cancer screening [8–12].

Nowadays, digital mammography (DM) is widely used and several software packages are available to measure breast density fully automatically on digital mammograms. Most packages report volumetric breast density measures, which are user-independent and developed with the intention to give an accurate and reproducible estimation of the amount of fibroglandular tissue in the breast using physics-based modeling based on information in the header of the raw digital images [13, 14]. This is in contrast to the aforementioned methods (BI-RADS and Cumulus) that measure the projected area of dense tissue on a mammogram. Density measured with automatic volumetric methods has also been examined in relation to breast cancer risk and results were found to be comparable to results from studies where BI-RADS or Cumulus were used to quantify breast density. In general, percentage dense volume (PDV) seemed to be a stronger breast cancer risk predictor than dense volume (DV) [15–18].Absolute dense volume represents the amount of fibroglandular tissue in the breast and is hypothesized to be positively associated with breast cancer risk as this is considered to be the actual tissue where breast cancers develop [19, 20]. Percent dense volume is the proportion of fibroglandular (dense) tissue in the breast. This has sometimes been suggested to be a stronger risk factor than absolute dense volume, which would indicate that the ratio between the two tissues is important, or that nondense volume, which is part of the denominator of percent dense volume, is a protective factor for breast cancer risk. In a large meta-analysis, Pettersson et al. previously studied the associations between both absolute and percentage dense area and breast cancer risk. Their results suggest that percentage dense area is a stronger risk indicator than absolute dense area, but some heterogeneity between study results remained [3]. By using automatically assessed volumetric breast density measures instead of projected area measures we hope to gain more insight into these ambiguities and to find the best measure to be used in breast cancer risk prediction.

In light of discussions on the need for supplemental screening after a negative mammogram, measures that indicate the risk of a tumor that is not detected at mammography screening (interval cancer) may be even more relevant. BI-RADS density and other area-based density measures have been shown to be strongly related to the risk of interval cancer [4, 21–26]. The relationship between automatic volumetric density methods and the risk of screen-detected and interval cancers, respectively, has not yet been studied. In this study, we used a fully automatic method to obtain several volumetric density measures, and assess their relationship with breast cancer risk. We distinguished between the risk of tumors that are detected at mammography (screen-detected cancers) or in the interval between two screening rounds (interval cancers), in a large cohort with digital mammograms from the population-based Dutch breast cancer screening program.

## Methods

### Study population

The study population consists of 52,814 women aged between 50 and 75 years who had one or more digital mammographic examinations at the Preventicon screening unit in Utrecht, the Netherlands, between 2003 and 2011. The Preventicon screening unit is one screening unit of the Foundation of Population Screening Mid-West screening region, which is one of the five screening regions of the Dutch biennial breast cancer screening program. All regions follow the same screening protocol. The Dutch program involves mammography only. By participating in the Dutch screening program, women consent to their data being used for evaluation and improvement of the screening, unless they have indicated otherwise.

### Data collection

In this breast cancer screening cohort, for each participant we collected the first unprocessed DM examination that was taken at the Preventicon screening unit between 2003 and 2011. All mammograms were acquired using Lorad Selenia DM systems (Hologic, Danbury, Conn.). If this was the participant’s first screening examination within the program, this examination included the two standard views, craniocaudal (CC) and mediolateral oblique (MLO). If this was not her first screening examination within the program, i.e. when she had undergone film screen mammography in earlier rounds, MLO was the routinely acquired view and the CC view was taken only when indicated (e.g. high breast density, visible abnormality). A CC view was present in 57% of the subsequent screening examinations. Information about follow up was obtained from the screening registration system. Women were followed until breast cancer diagnosis (event), death or until 2 years after the last available mammogram, whichever came first. Information on breast cancer development was obtained from the screening registration system and through linkage with the Netherlands Cancer Registry. This also included information on the mode of detection: through screening mammography (screen-detected cancer) or through diagnosis in the 24 months screening interval after a negative screening mammogram (interval cancer). Interval cancers diagnosed more than 24 months after a negative screening mammogram were not included for analysis. Within the screening program, information on risk factors other than age is not collected.

### Volumetric mammographic density assessment

Absolute dense volume (DV), percentage dense volume (PDV) and absolute nondense volume (NDV) were automatically assessed from unprocessed mammograms of the left and right breasts, using Volpara density (version 1.5.0, Matakina, Wellington, New Zealand). Although normally both MLO and CC views would be used for calculating volumetric density measures with Volpara, here we used the mean of only the left and right MLO views for the risk analyses, since this is the routinely acquired view and CC views were not available for all women. In this way we ensured that breast density was assessed in the exact same way in all participants. Volpara density grades (VDGs) were constructed based on this average PDV using the standard Volpara cutoff points from version 1.5.0 (VDG1: 0% ≤ PDV <4.5%, VDG2: 4.5% ≤ PDV <7.5%, VDG3: 7.5% ≤ PDV <15.5%, VDG4: PDV ≥15.5%). The VDGs are designed to mimic the fourth edition of the BI-RADS breast density categories. We chose to use this edition, because it is most comparable to what has been used in previous papers.

### Statistical analysis

Descriptive statistics of age and breast measures (DV, PDV, NDV and VDG) were determined. Continuous breast measures were transformed using the natural logarithm (ln) to obtain normal distributions. Pearson correlation coefficients for correlation between different breast measures and between breast measures and age were determined. We also assessed the correlation between breast measures on the MLO and CC views among women who had both views available, and on the MLO views of the left and right breasts.

We examined the associations between the different breast density measures and breast cancer risk by calculating hazard ratios (HR) and their 95% confidence intervals (95% CI) using Cox proportional hazards analyses for quartiles (quartile cutoffs were determined based on the distribution of the different density measures in the whole cohort at baseline) and continuous measures (per standard deviation (SD) increase of the ln-transformed measure) of DV and PDV. We also determined associations between breast cancer risk and two additional breast density measures: (1) VDG categories (Volpara BI-RADS) and (2) a categorized DV variable in which the cutoff points were created as to obtain the same group sizes as with the VDG categories (VDG-like categories).

To minimize the number of breast cancer cases in the model that were diagnosed based on the same mammogram as was used for breast density assessment, we excluded all screen-detected breast cancer cases of women diagnosed on the basis of their first digital screening examination.

Cox proportional hazards analyses were used to examine the association between breast density and breast cancer risk. Age was used as the underlying time scale with entry and exit time defined as age at time of the first available DM and age at breast cancer diagnosis, death or censoring 2 years after the last DM before 1 January 2012. In this way, the confounding effect of age was taken into account. Two models were constructed. The first model only contained one of the density measures. The second model was adjusted for NDV in case the model included quartiles, categories or continuous measures of DV. We found that DV and NDV volume were positively correlated (Pearson correlation = 0.50). By adjusting for NDV we attempted to determine the independent effect of DV on breast cancer risk, without the interference of NDV. Models containing a PDV measure (including VDG) were not adjusted for NDV, since NDV is already part of PDV (PDV = DV/(DV + NDV)). The proportional hazards assumption was evaluated by Schoenfeld residual plots and log-minus-log plots, and the assumption was not violated. To examine the presence of a linear trend in HRs over the quartiles and categories of breast measures, quartile and category variables were added to the models as continuous variables.

To examine the association between the different density measures and screen-detected or interval cancers and to test whether the association was statistically significantly different between density measures and screen-detected versus interval cancers, the method of Lunn and McNeil was used for competing risk analysis [27, 28]. Cox proportional hazards analysis was used for this method, with time of follow up as the underlying time scale. Age at time of first available DM was added as a covariate in the models to take the confounding effect of age into account.

A bootstrap method was used to determine whether measures based on PDV or DV were stronger breast cancer risk factors. This was done for VDG versus VDG-like categories, quartiles of PDV versus quartiles of DV and continuous PDV versus continuous DV. The differences between coefficients for models containing a PDV or DV measure were determined within 2000 bootstrap samples. A difference of 0 indicates that there is no difference between the effect of DV and PDV on breast cancer risk. A 95% CI that does not include 0 means that one measure is statistically significantly more strongly related to breast cancer risk than the other measure.

In addition, ﻿a sensitivity analysis was performed where we only used the contralateral MLO view for breast cancer cases and for non-breast cancer cases the MLO view of a random chosen side, with the same left and right distribution as that of the breast cancer cases.

For the main analysis both invasive and in situ breast cancers were used. In a sensitivity analysis, we only took invasive breast cancers into account for data analysis.

For all breast density measures, we compared the fourth category or quartile with the first. Nowadays, often the second category is used as the reference category, as BI-RADS breast density category 2 is the most prevalent category among women of breast cancer screening age. To be able to compare with other studies, we also present results using the second category or quartile of each breast density measure as reference category. Statistical analyses were performed using SPSS version 22 and R version 3.2.0.

## Results

After excluding cases of screen-detected breast cancer, diagnosed on the basis of the first available digital screening examination, 523 breast cancers, including 299 screen-detected and 224 interval breast cancers were used for analyses. At the first digital screening mammogram the median age was 56 years (IQR 51–63) in women who did not develop breast cancer (N = 52,291), 59 years (IQR 54–64) in women who were diagnosed with screen-detected breast cancer and 55 years (IQR 50–62) in women diagnosed with interval breast cancer during follow up. The median total number of screening rounds in which women participated (analog (if applicable) and digital together) was 5 (IQR 2–8), 7 (IQR 4–9) and 4 (IQR 2–7) in women who were not diagnosed with breast cancer, were diagnosed with a screen-detected breast cancer and were diagnosed with interval cancer, respectively. The median follow-up time after the first digital mammogram until breast cancer diagnosis (event), death or end of follow up, was 4.2 years (IQR 2.0–6.2), 3.8 years (IQR 2.1–4.3) and 2.3 (IQR 1.3–4.0), in women with no diagnosis of breast cancer, screen-detected breast cancer and interval breast cancer, respectively (Table 1).

**Table 1:** Characteristics of 52,814 women undergoing digital mammography (first time) between 2003 and 2011

	No breast cancer (*N* = 52,291)		Screen-detected cancer (*N* = 299)		Interval cancer (*N* = 224)	
Age (years)^a^, median (IQR)	56	(51 ; 63)	59	(54 ; 64)	55	(50 ; 62)
Number of screening rounds participated in, median (IQR)	5	(2 ; 8)	7	(4 ; 9)	4	(2 ; 7)
Follow up (years)^b^, median (IQR)	4.2	(2.0 ; 6.2)	3.8	(2.1 ; 4.3)	2.3	(1.3 ; 4.0)
VDG categories, *n* (%)						
Category 1	10,458	(20.0)	46	(15.4)	12	(5.4)
Category 2	21,276	(40.7)	143	(47.8)	72	(32.1)
Category 3	15,856	(30.3)	89	(29.8)	100	(44.6)
Category 4	4,701	(9.0)	21	(7.0)	40	(17.9)
Continuous density measures, median (IQR)						
Dense volume (cm^3^)^a^	57.7	(42.8 ; 78.8)	63.2	(49.0 ; 83.5)	67.7	(48.9 ; 93.2)
Percent dense volume (%)^a^	6.4	(4.8 ; 9.8)	6.5	(5.0 ; 9.4)	8.5	(6.2 ; 13.6)
Nondense volume (cm^3^)^a^	805.0	(518.8 ; 1183.7)	929.1	(585.6 ; 1298.7)	720.3	(469.8 ; 1030.9)
Total breast volume (cm^3^)^a^	866.8	(573.9 ; 1256.8)	994.0	(648.6 ;1376.5)	797.5	(531.8 ; 1105.8)

*VDG* Volpara density grade

^a^At first digital screening mammogram

^b^Women were followed until breast cancer diagnosis (event), till death or till 2 years after the last available mammogram, whichever came first

PDV was strongly negatively correlated with NDV (-0.70, *p* < 0.01)) and positively correlated with DV (0.27, *p* < 0.01). DV was positively correlated with NDV (0.50, *p* < 0.01). DV and PDV were both negatively correlated with age (-0.16 and -0.29, respectively, *p* < 0.01), whereas NDV was positively correlated with age (0.14, *p* < 0.01). There was strong correlation between breast measures of the left and right breast and between CC and MLO views. Correlations between the MLO views of the left and right breast for DV, PDV and NDV was 0.86, 0.91 and 0.98, respectively (N = 52,410 and *p* < 0.01 for all three). Correlation between MLO and CC views of the right breast was 0.86, 0.90 and 0.97 DV, PDV and NDV, respectively (N = 38,997 and *p* < 0.01 for all three).

### Association between breast density measures and breast cancer risk (total)

**Fig. 1:**
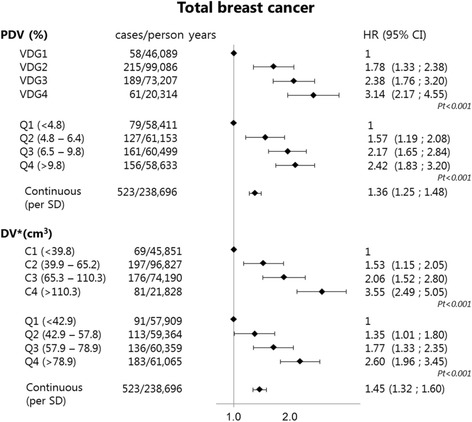
Associations between mammographic measures and breast cancer risk. In the cox proportional hazards analyses age was used as the underlying time scale. *Pt* p-trend: this was determined by adding the categorical measures as a continuous measure to the model, *PDV* percentage dense volume, *DV* dense volume, *Per SD* per standard deviation, *VDG* Volpara density grade, *Q* quartile, *C* category. *Absolute dense volume (DV) measures were adjusted for nondense (breast fat) volume

In Fig. 1 and Table 2 it is shown that all breast density measures (VDG, quartiles of PDV, continuous PDV, VDG-like categories, quartiles of DV and continuous DV) were positively associated with breast cancer risk, with HR 3.14 (95% CI 2.17–4.55) for VDG category 4 compared to category 1 and HR 3.55 (95% CI 2.49–5.05) for VDG-like category 4 compared to category 1, and HR 2.42 (95% CI 1.83–3.20) for PDV and 2.60 (95% CI 1.96–3.45) for DV quartile 4 compared to quartile 1. The risk estimates of PDV and DV measures were not statistically significantly different from one another (Table 3).

**Table 2:** Association between mammographic measures and **breast cancer risk**

	Cases/person years	HR_1_ (95% CI)	HR_2_ (95% CI)	HR_invasive_ (95% CI)
Volpara density grades				
VDG1	58/46,089	ref	NA	ref
VDG2	215/99,086	1.78 (1.33 ; 2.38)	NA	1.89 (1.38 ; 2.58)
VDG3	189/73,207	2.38 (1.76 ; 3.20)	NA	2.53 (1.83 ; 3.49)
VDG4	61/20,314	3.14 (2.17 ; 4.55)	NA	3.54 (2.39 ; 5.24)
*P trend*		<0.001		<0.001
Percent dense volume (in quartiles)				
Q1 (<4.8%)	79/58,411	ref	NA	ref
Q2 (4.8–6.4%)	127/61,153	1.57 (1.19 ; 2.08)	NA	1.61 (1.19 ; 2.18)
Q3 (6.4–9.8%)	161/60,499	2.17 (1.65 ; 2.84)	NA	2.33 (1.74 ; 3.11)
Q4 (>9.8%)	156/58,633	2.42 (1.83 ; 3.20)	NA	2.52 (1.87 ; 3.40)
*P trend*		<0.001		<0.001
Percent dense volume (per SD increase)^#^	523/238,696	1.36 (1.25 ; 1.48)	NA	1.38 (1.26 ; 1.51)
Dense volume (VDG-like categories)				
C1 (<39.8 cm^3^)	69/45,851	ref	ref	ref
C2 (39.9– 65.2 cm^3^)	197/96,827	1.37 (1.04 ; 1.80)	1.53 (1.15 ; 2.05)	1.69 (1.24 ; 2.30)
C3 (65.3– 110.1 cm^3^)	176/74,190	1.66 (1.26 ; 2.20)	2.06 (1.52 ; 2.80)	2.27 (1.63 ; 3.14)
C4 (>110.1 cm^3^)	81/21,828	2.79 (2.02 ; 3.86)	3.55 (2.49 ; 5.05)	3.66 (2.50 ; 5.36)
*P trend*		<0.001	<0.001	<0.001
Dense volume (in quartiles)				
Q1 (<42.9 cm^3^)	91/57,909	ref	ref	ref
Q2 (42.9–7.8 cm^3^)	113/59,364	1.22 (0.93 ; 1.61)	1.35 (1.01 ; 1.80)	1.47 (1.08 ; 1.99)
Q3 (57.9–78.9 cm^3^)	136/60,359	1.48 (1.13 ; 1.93)	1.77 (1.33 ; 2.35)	1.92 (1.42 ; 2.60)
Q4 (>78.9 cm^3^)	183/61,065	2.08 (1.61 ; 2.68)	2.60 (1.96 ; 3.45)	2.71 (2.01 ; 3.67)
*P trend*		<0.001	<0.001	<0.001
Dense volume (per SD increase)^#^	523/238,696	1.34 (1.23 ; 1.46)	1.45 (1.32 ; 1.60)	1.45 (1.32 ; 1.60)

*HR*_*1*_ Cox proportional hazards analysis where age was used as the underlying time scale, *HR*_*2*_ dense volume HR’s are adjusted for nondense volume (quartiles), *HR*_*invasive*_ Cox proportional hazards analysis with only invasive breast cancers, where age was used as the underlying time scale. Dense volume HRs were adjusted for nondense volume (quartiles), *NA* Not applicable

*P* trend was determined by adding the categorical measures as a continuous measure into the model

^#^Continuous measures were natural-logarithm-transformed to establish normal distributions

**Table 3:** Percentage dense volume versus dense volume measures in relation to breast cancer risk (bootstrap analysis results)

Total breast cancers	Significance of the difference between coefficients in PDV and DV models	Difference between coefficients in PDV and DV models	95% CI of difference between coefficients in PDV and DV models
VDG vs VDG-like categories	Non significant	C2: 0.15	(-0.25 ; 0.56)
		C3: 0.15	(-0.23 ; 0.52)
		C4: -0.11	(-0.51 ; 0.27)
Quartiles PDV vs DV	Non significant	Q2: 0.16	(-0.24 ; 0.54)
		Q3: 0.21	(-0.17 ; 0.57)
		Q4: -0.07	(-0.37 ; 0.24)
Continuous PDV vs DV	Non significant	-0.07	(-0.15 ; 0.01)
Screen-detected cancers			
VDG vs VDG-like categories	Non significant	C2: -0.03	(-0.56 ; 0.46)
		C3: -0.17	(-0.64 ; 0.28)
		C4: -0.49	(-1.08 ; 0.07)
Quartiles PDV vs DV	Non significant	Q2: -0.14	(-0.65 ; 0.32)
		Q3: -0.14	(-0.62 ; 0.34)
		**Q3: -0.49**	**(-0.90 ; -0.12)**
Continuous PDV vs DV	**Significant, stronger for DV**	**-0.13**	**(-0.24 ; -0.03)**
Interval cancers			
VDG vs VDG-like categories	Non significant (except for C2 and C3: stronger for PDV)	**C2: 0.73**	**(0.01 ; 1.56)**
		**C3: 0.92**	**(0.26 ; 1.68)**
		C4: 0.58	(-0.06 ; 1.28)
Quartiles PDV vs DV	**Significant, stronger for PDV**	**Q2: 0.90**	**(0.21 ; 1.60)**
		**Q3: 1.02**	**(0.40 ; 1.66)**
		**Q3: 0.77**	**(0.26 ; 1.34)**
Continuous PDV vs DV	non significant	0.01	(-0.11 ; 0.13)

*VDG* Volpara density grade, *PDV* percentage dense volume, *DV* dense volume

Significant: none of the bootstrap 95% CIs for differences between quartile (Q)2, Q3, or Q4 (or category (C)2, C3, or C4) contain zero, otherwise they were non-significant

Bold text means that the difference between coefficients in PDV an DV models are significant

The HRs for continuous measures of PDV and DV were 1.36 (95% CI 1.25–1.48) and 1.45 (95% CI 1.32–1.60), respectively, for one standard deviation increase in the ln-transformed continuous measure (not statistically significantly different from one another (Table 3)).

### Association between breast density measures and **screen-detected breast cancer** risk

**Fig. 2:**
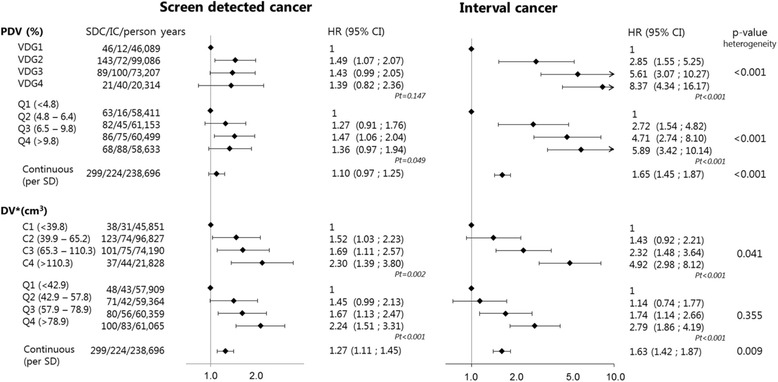
Associations between mammographic measures and risk of screen-detected breast cancer (*SDC*) or interval breast cancer (*IC*). The Lunn and McNeil method for competing risk analysis was used. *Pt p* trend: this was determined by adding the categorical measures as a continuous measure into the model, *PDV* percentage dense volume, *DV* dense volume, *Per SD* per standard deviation, *VDG* Volpara density grade, *Q* quartile, *C* VDG-like category. *Absolute dense volume measures were adjusted for nondense (breast fat) volume

Figure 2 and Table 4 show the association between different breast density measures and the risk of screen-detected breast cancer. The measures based on DV (VDG-like categories, quartiles of DV and continuous DV) were positively associated with the risk of screen-detected breast cancer. The associations between PDV measures (VDG, quartiles of PDV and continuous PDV) and screen-detected breast cancer risk were increased, but were not statistically significant or were borderline significant.

**Table 4:** Association between mammographic measures and **screen-detected breast cancer risk** - Lunn and McNeil

	Cases/person years	HR_1_ (95% CI)	HR_2_ (95% CI)	HR_invasive_ (95% CI)
Volpara density grades				
VDG1	46/46,089	ref	NA	ref
VDG2	143/99,086	1.49 (1.07 ; 2.07)	NA	1.53 (1.07 ; 2.21)
VDG3	89/73,207	1.43 (0.99 ; 2.05)	NA	1.41 (0.94 ; 2.10)
VDG4	21/20,314	1.39 (0.82 ; 2.36)	NA	1.41 (0.79 ; 2.54)
*P trend*		0.147		0.219
Percent dense volume (in quartiles)				
Q1 (<4.8%)	63/58,411	ref	NA	ref
Q2 (4.8–6.4%)	82/61,153	1.27 (0.91 ; 1.76)	NA	1.27 (0.89 ; 1.81)
Q3 (6.4–9.8%)	86/60,499	1.47 (1.06 ; 2.04)	NA	1.52 (1.06 ; 2.17)
Q4 (>9.8%)	68/58,633	1.36 (0.97 ; 1.94)	NA	1.27 (0.86 ; 1.89)
*P trend*		0.049		0.119
Percent dense volume (per SD increase)^#^	299/238,696	1.10 (0.97 ; 1.25)	NA	1.08 (0.94 ; 1.24)
Dense volume (VDG-like categories)				
C1 (<39.8 cm^3^)	38/45,851	ref	ref	ref
C2 (39.9–65.2 cm^3^)	123/96,827	1.55 (1.08 ; 2.23)	1.52 (1.03 ; 2.23)	1.76 (1.14 ; 2.72)
C3 (65.3–110.1 cm^3^)	101/74,190	1.76 (1.21 ; 2.56)	1.69 (1.11 ; 2.57)	1.99 (1.24 ; 3.17)
C4 (>110.1 cm^3^)	37/21,828	2.40 (1.52 ; 3.79)	2.30 (1.39 ; 3.80)	2.03 (1.13 ; 3.67)
*P trend*		<0.001	0.002	0.014
Dense volume (in quartiles)*				
Q1 (<42.9 cm^3^)	48/57,909	ref	ref	ref
Q2 (42.9–57.8 cm^3^)	71/59,364	1.46 (1.01 ; 2.10)	1.45 (0.99 ; 2.13)	1.62 (1.06 ; 2.48)
Q3 (57.9–78.9 cm^3^)	80/60,359	1.67 (1.16 ; 2.39)	1.67 (1.13 ; 2.47)	1.86 (1.21 ; 2.87)
Q4 (>78.9 cm^3^)	100/61,065	2.22 (1.57 ; 3.14)	2.24 (1.51 ; 3.31)	2.31 (1.49 ; 3.58)
*P trend*		<0.001	<0.001	<0.001
Dense volume (per SD increase)^#^	299/238,696	1.27 (1.14 ; 1.43)	1.27 (1.11 ; 1.45)	1.22 (1.05 ; 1.41)

*HR*_*1*_ Lunn and McNeil method was used for competing risk analysis, *HR*_*2*_ dense volume HR’s were additionally adjusted for nondense volume (quartiles), *HR*_*invasive*_ Cox proportional hazards analysis with only invasive breast cancers, where age was used as the underlying time scale. Dense volume HRs were adjusted for nondense volume (quartiles). *P* trend was determined by adding the categorical measures as a continuous measure into the model, *NA* Not applicable

^#^Continuous measures were natural-logarithm-transformed to establish normal distributions

The HRs were 2.30 (95% CI 1.39–3.80) and 1.39 (95% CI 0.82–2.36) for VDG-like category and VDG category 4 compared to category 1, respectively and were 2.24 (95% CI 1.51–3.31) and 1.36 (95% CI 0.97–1.94) for the highest compared to the lowest quartiles of DV and PDV, respectively. The HRs were 1.27 (95% CI 1.14–1.43) and 1.10 (0.97–1.25) for continuous measures of DV and PDV, respectively. DV, measured continuously, had a significantly stronger association with screen-detected breast cancer risk than continuously measured PDV (Table 3). Although DV was significantly associated with screen-detected breast cancer risk, and PDV was not, the risk estimates for VDG versus VDG-like categories and PDV versus DV quartiles in relation to screen-detected breast cancer risk were not statistically significantly different (Table 3).

### Association between breast density measures and **interval breast cancer** risk

The association between breast density measures and interval breast cancer risk is presented in Fig. 2 and Table 5. All breast density measures were statistically significantly associated with increased risks of interval breast cancer. The HRs were 4.92 (95% CI 2.98–8.12) and 8.37 (95% CI 4.34–16.17) for VDG-like category and VDG category 4 compared to category 1, respectively and 2.79 (95% CI 1.86–4.19) and 5.89 (95% CI 3.42–10.14) for the highest compared to the lowest quartiles of DV and PDV, respectively. Quartiles of PDV were more strongly associated with interval breast cancer risk than quartiles of DV, and this difference was statistically significant (Table 3). The difference in effect between VDG versus VDG-like-categories was not statistically significantly different. The continuous measures of PDV and DV were also positively associated with interval breast cancer risk, with HRs of 1.65 (95% CI 1.45–1.87) and 1.63 (95% CI 1.42–1.87) for one standard deviation increase in the ln-transformed continuous measure (not significant (Table 3)).

**Table 5:** Association between mammographic measures and **interval breast cancer risk** - Lunn and McNeil

	Cases/person years	HR_1_ (95% CI)	HR_2_ (95% CI)	HR_invasive_ (95% CI)
Volpara density grades				
VDG1	12/46,089	ref	NA	ref
VDG2	72/99,086	2.85 (1.55 ; 5.25)	NA	3.03 (1.61 ; 5.73)
VDG3	100/73,207	5.61 (3.07 ; 10.27)	NA	6.01 (3.20 ; 11.27)
VDG4	40/20,314	8.37 (4.34 ; 16.17)	NA	9.30 (4.71 ; 18.37
*P trend*		<0.001		<0.001
Percent dense volume (in quartiles)				
Q1 (<4.8%)	16/58,411	ref	NA	ref
Q2 (4.8–6.4%)	45/61,153	2.72 (1.54 ; 4.82)	NA	2.78 (1.55 ; 5.01)
Q3 (6.4–9.8%)	75/60,499	4.71 (2.74 ; 8.10)	NA	4.99 (2.86 ; 8.72)
Q4 (>9.8%)	88/58,633	5.89 (3.42 ; 10.14)	NA	6.22 (3.56 ; 10.89)
*P trend*		<0.001		<0.001
Percent dense volume (per SD increase)^#^	224/238,696	1.65 (1.45 ; 1.87)	NA	1.68 (1.48 ; 1.91
Dense volume (VDG-like categories)				
C1 (<39.8 cm^3^)	31/45,851	ref	ref	ref
C2 (39.9–65.2 cm^3^)	74/96,827	1.13 (0.75 ; 1.73)	1.43 (0.92 ; 2.21)	1.48 (0.95 ; 2.30)
C3 (65.3–110.1 cm^3^)	75/74,190	1.51 (0.99 ; 2.29)	2.32 (1.48 ; 3.64)	2.32 (1.47 ; 3.67)
C4 (>110.1 cm^3^)	44/21,828	3.01 (1.89 ; 4.79)	4.92 (2.98 ; 8.12)	5.24 (3.16 ; 8.70)
*P trend*		<0.001	<0.001	<0.001
Dense volume (in quartiles)*				
Q1 (<42.9 cm^3^)	43/57,909	ref	ref	ref
Q2 (42.9–57.8 cm^3^)	42/59,364	0.95 (0.62 ; 1.46)	1.14 (0.74 ; 1.77)	1.22 (0.78 ; 1.90)
Q3 (57.9–78.9 cm^3^)	56/60,359	1.25 (0.84 ; 1.87)	1.74 (1.14 ; 2.66)	1.83 (1.19 ; 2.81)
Q4 (>78.9 cm^3^)	83/61,065	1.83 (1.26 ; 2.66)	2.79 (1.86 ; 4.19)	2.89 (1.91 ; 4.38)
*P trend*		<0.001	<0.001	<0.001
Dense volume (per SD increase)^#^	224/238,696	1.39 (1.22 ; 1.59)	1.63 (1.42 ; 1.87)	1.65 (1.44 ; 1.90)

*HR*_*1*_ Lunn and McNeil method was used for competing risk analysis, *HR*_*2*_ dense volume HRs were adjusted for nondense volume (quartiles), *HR*_*invasive*_ Cox proportional hazards analysis with only invasive breast cancers, where age was used as the underlying time scale. Dense volume HRs were adjusted for nondense volume (quartiles). *P* trend was determined by adding the categorical measures as a continuous measure into the model, *NA* Not applicable

^#^Continuous measures were natural-logarithm-transformed to establish normal distributions

For all breast measures (except quartiles of DV (*p* = 0.355)) the risk in relation to interval breast cancer was statistically significantly stronger than that in relation to screen-detected breast cancer (Fig. 2). The associations between percentage dense volume and interval breast cancer risk seemed somewhat stronger than the associations between absolute dense volume and interval breast cancer risk, although this difference between percentage and absolute dense volume was not consistently statistically significant (Table 3).

Of the 523 breast cancers in our study, 56 (11%) were in situ, and 466 (89%) were invasive breast cancers. For one case of breast cancer, information on invasiveness was missing. Of the interval cancers, 218 (97%) were invasive breast cancers compared to 83% of screen-detected cancers. Results restricted to invasive breast cancers are presented in Tables 2, 4 and 5. In general, the effects for invasive breast cancer were similar to those for invasive and in situ cancers combined.

Our results did not change when we only used the contralateral MLO view for breast cancer cases and the MLO view of a random chosen side for non-breast cancer cases (instead of the mean of the left and right view) for breast density measurements in relation to breast cancer risk (data not shown).

For reference and for comparison with other papers, Additional file 1: Figure S1 and Additional file 2: Figure S2 are presented with the second instead of the first breast density categories as the reference category.

## Discussion

In this study we found that automatically assessed volumetric breast density measures are positively associated with breast cancer risk and in particular with the risk of interval cancer. The difference in effect on interval and screen-detected cancers appeared slightly more pronounced for the percentage dense volume measures than for absolute dense volume measures. The VDG categories that are comparable to the BI-RADS categories had the highest risk estimates for association with interval breast cancers.

To our knowledge the relationship between volumetric breast density and risk of screen-detected and interval cancers, respectively, has not been studied separately before, but there are several studies on the association between volumetric breast density, determined on digital mammograms, and total breast cancer risk. An overview of their design and results is given in Additional file 3: Table S1. Four of the five presented studies examined both absolute and percentage dense volume in relation to breast cancer risk. In general, the risk estimates for percentage dense volume are somewhat higher than for absolute dense volume [15–18]. Although our risk estimates for dense volume and percentage dense volume are in line with those of the studies presented in Additional file 3: Table S1, our percentage dense volume risk estimates (VDG, PDV) seem to be slightly lower than in the other studies [15–18]. This could be explained by the fact that we do not have information on body mass index (BMI) or other risk factors in the routine screening program and therefore could not adjust for these risk factors. In particular the adjustment for BMI is known to increase the risk estimates for percent density measures. Despite this lack of adjustment for BMI, still the percent dense volume measures in our study appear to be more strongly related to the risk of interval cancer than the absolute dense volume measures.

As mentioned, no other studies have examined the ability of automated volumetric breast density assessment methods to identify women with a high risk of interval breast cancer. However, from a breast cancer screening perspective, this is important to know, since women with a high risk of interval breast cancer might benefit most from supplemental screening. Area-based breast density measures, such as BI-RADS density and the semi-automatic Cumulus thresholding method have been studied in relation to interval cancer risk. In these studies density was found to be more strongly related to the risk of an interval cancer than to the risk of a screen-detected cancer [4, 22–26, 29]. Boyd et al. (2014) and Krishnan et al. (2016) were the only two studies of both absolute dense area and percent dense area in relation to the mode of breast cancer detection. They too found that breast density was significantly more strongly related to the risk of interval breast cancers than of screen-detected breast cancers. This difference in effect seemed stronger for percentage dense area than for absolute dense area, although this was not formally tested [22, 29].

Likewise, in our study percentage dense volume seems to be a better marker for interval breast cancer risk than dense volume. When looking at total breast cancer risk, this difference between percentage dense volume and absolute dense volume seems less pronounced. An explanation for this finding could be that the amount of dense tissue is etiologically relevant for breast cancer risk, and that when this dense tissue is occupying the larger part of the breast volume, these women will run a higher risk that a tumor will be masked at mammography and later diagnosed as interval cancer. If on the other hand, this same amount of dense tissue is accompanied by a large amount of fat tissue, large parts of the mammogram may still be well “readable” for cancer detection, and the probability that this tumor is detected at the screening examination will become higher.

Besides masking, another possible explanation for the relatively larger number of interval breast cancers in women with dense breasts is tumor aggressiveness. It is possible that tumors in dense breasts are more aggressive (grow faster) than tumors in nondense breasts. However, although some studies report that breast density is more strongly related to estrogen receptor (ER)-negative tumors than to ER-positive tumors, this was not confirmed in other studies [30–34].

When interpreting our results, it should be kept in mind that we excluded prevalent breast cancer cases (screen-detected cancers diagnosed as a result of the first digital screening examination). The proportion of interval cancers is therefore larger (43%) in the current study than in the Dutch biennial screening program (26%). As interval cancers are relatively more common in dense breasts, the association between breast density and total breast cancer risk is probably somewhat overestimated. However, this is not the case for analyses separated for screen-detected and interval breast cancers.

In most European countries women are screened from the age of 50 years. In the USA most women are screened from the age of 40 years. In addition, biennial screening is common in European countries, while in the USA many women are screened yearly. We therefore looked at interval cancers diagnosed within the first year after a screening mammogram. We observed that 22% of the interval cancers in the group with the lowest breast density was found in that first year, while this was 60% in the group with the highest breast density, indicating an even stronger association between breast density and risk of interval breast cancer within the first year after screening.

A limitation of our study is that we could not compare the association between the automated volumetric density measurements and breast cancer risk with those of other commonly used breast density measurements such as BI-RADS. The reason for this is that BI-RADS density readings are not routinely performed in the Dutch breast cancer screening program. Previous studies directly comparing automated density measuring methods to BI-RADS density readings and other breast density measurements in relation to breast cancer risk identified similar positive associations for all density measurements, although they may differ in their classification of which women have dense breasts and which women have not [15–18, 35, 36]. It remains as yet unknown which method most accurately reflects true density [17]. The method that we used in our study has been validated against MRI measurements of the fibroglandular tissue volume and by its fully automatic nature this gives objective and reproducible density measurements [13]. Although BI-RADS density is known to be strongly associated with breast cancer and interval breast cancer risk, the inter-reader reproducibility using this method is moderate [8–12]. A critical issue now in the discussion of the value of supplemental imaging for women with dense breasts is that it may depend on the radiologist who interprets the mammograms as to whether a woman is classified as having dense breasts or not [12].

Another limitation is that at the time the mammographic examinations were performed, the CC view was not a routine view in our screening program. The CC view was only taken when women had their first screening examination, or on indication, e.g. because of high breast density or suspect lesions. Although for robustness of volumetric breast density estimates it may have been preferable to calculate density on MLO and CC views, in our study we did this on the MLO view only, to prevent effect estimates from being biased by the selective availability of the CC views.

A final limitation is that we only had information about age as an important confounder. Therefore, adjustment of the risk estimates for other confounders was not possible. The inability to adjust for parity and other reproductive factors may have overestimated the risk estimates somewhat both for dense volume and percent dense volume. Not adjusting for BMI is expected to have led to an underestimation of the risk estimate of percentage dense volume in particular, but all in all, the risk estimates for total breast cancer risk are quite comparable to what has been published in other papers on volumetric breast density [15–18, 37]. Besides that, our study could be seen as an example for other screening programs where often little or no information is available on risk factors other than age.

Strengths of our study are the large cohort, the availability of unprocessed digital data for all its participants and its follow up since 2003 when digital mammography screening was introduced in the Netherlands. Due to linkage with the Netherlands Cancer Registry, which covers all residents of the Netherlands and is more than 95% complete, we have virtually complete information not only on screen-detected, but also on interval breast cancers.

## Conclusion

We found that automated breast density measures can be used for breast cancer risk assessment and in particular, for the risk of a tumor that is not detected at the breast cancer screening. Automated measures are especially useful in programs where BI-RADS is not routinely determined or to improve reproducibility.

## Additional files

Additional file 1: Figure S1. Associations between mammographic density measures and breast cancer risk (second category used as reference). In the Cox proportional hazards analyses age was used as the underlying time scale. *Pt p* trend: this was determined by adding the categorical measures as a continuous measure into the model, *PDV* percentage dense volume, *DV* dense volume, *Per SD* per standard deviation. *Absolute dense volume measures are adjusted for nondense (breast fat) volume. (DOCX 185 kb)

Additional file 2: Figure S2. Mammographic measures and screen-detected, and interval breast cancer risk (second category used as reference). Lunn and McNeil method was used for competing risk analysis. *Pt p* trend: this was determined by adding the categorical measures as a continuous measure into the model, *PDV* percentage dense volume, *DV* dense volume, *Per SD* per standard deviation, *SDC* screen-detected cancer, *IC* interval cancer. *Absolute dense volume measures are adjusted for nondense (breast fat) volume. (DOCX 890 kb)

Additional file 3: Table S1. Overview of associations between volumetric breast density measures (measured with Volpara) and breast cancer risk. Overview of studies that also determined the association between Volpara density grade categories and breast cancer risk. (DOCX 23 kb)

## Abbreviations

BI-RADS: Breast Imaging - Reporting and Data System
BMI: Body mass index
CC: Craniocaudal
DM: Digital mammography
DV: Dense volume
ER: Estrogen receptor
HR: Hazard ratio
IQR: Interquartile range
Ln: Natural logarithm
MLO: Mediolateral oblique
MRI: Magnetic resonance imaging
NDV: Nondense (breast fat) volume
PDV: Percentage dense volume
Q: Quartile
SD: Standard deviation
VDGs: Volpara density grades
