# Supplementary figures and images for: The effect of volumetric breast density on the risk of screen-detected and interval breast cancers: a cohort study
Source: Breast Cancer Res. 2017 Jun 5;19:67. doi: 10.1186/s13058-017-0859-9 (PMC5460501; doi:10.1186/s13058-017-0859-9)

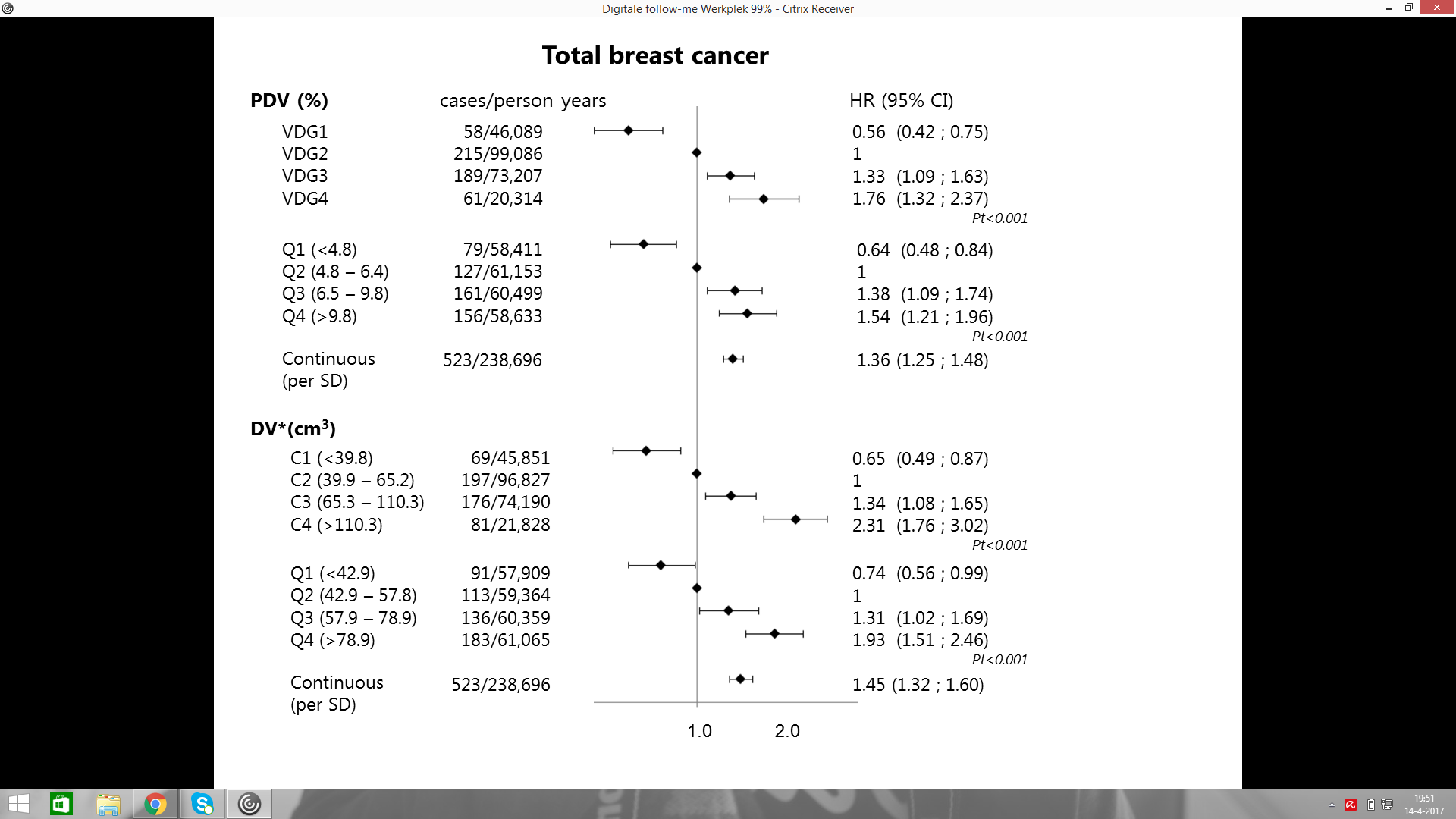

Supplement: Supplementary file 1 — Associations between mammographic density measures and breast cancer risk (second category used as reference). In the Cox proportional hazards analyses age was used as the underlying time scale. Pt p trend: this was determined by adding the categorical measures as a continuous measure into the model, PDV percentage dense volume, DV dense volume, Per SD per standard deviation. *Absolute dense volume measures are adjusted for nondense (breast fat) volume. (DOCX 185 kb) [file 13058_2017_859_MOESM1_ESM.docx]

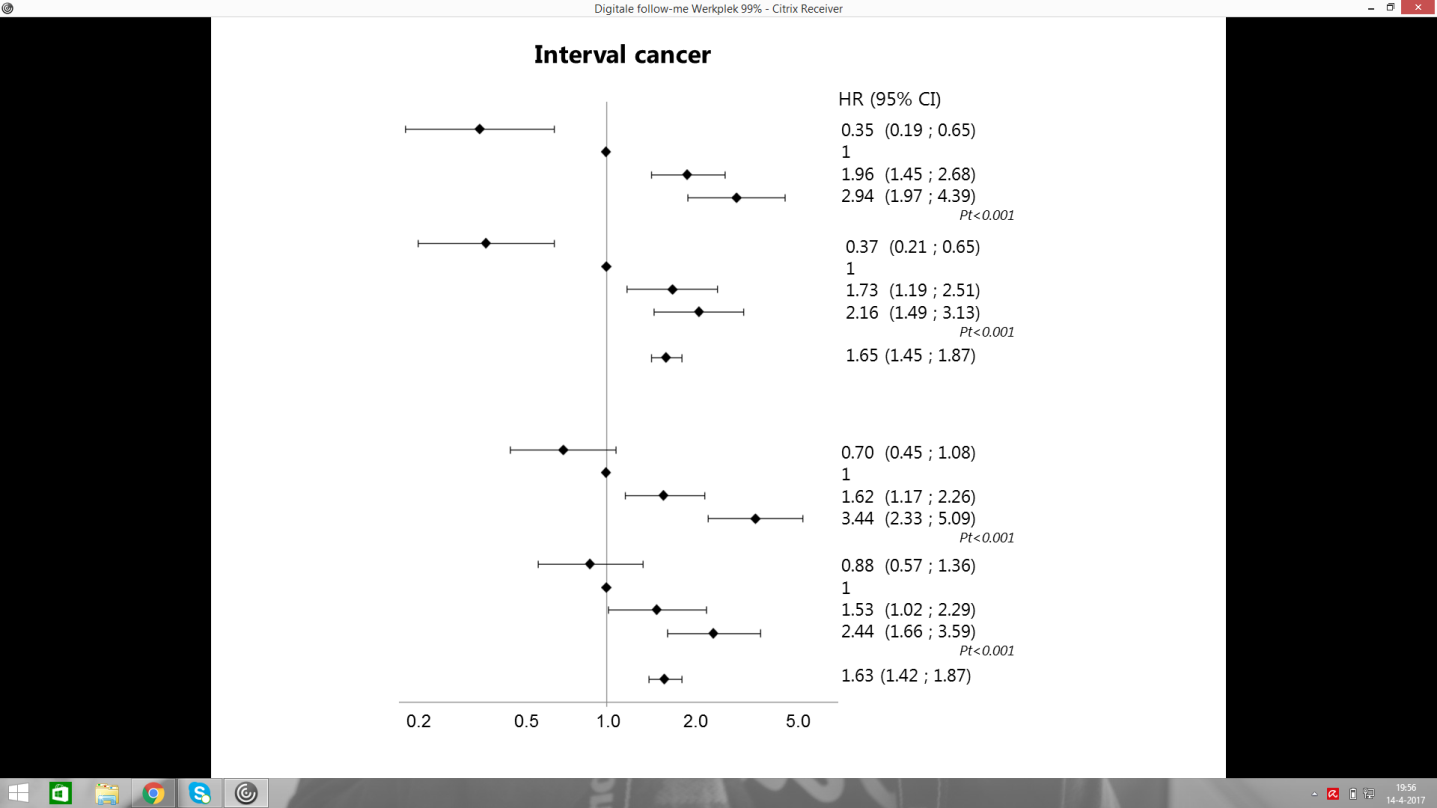

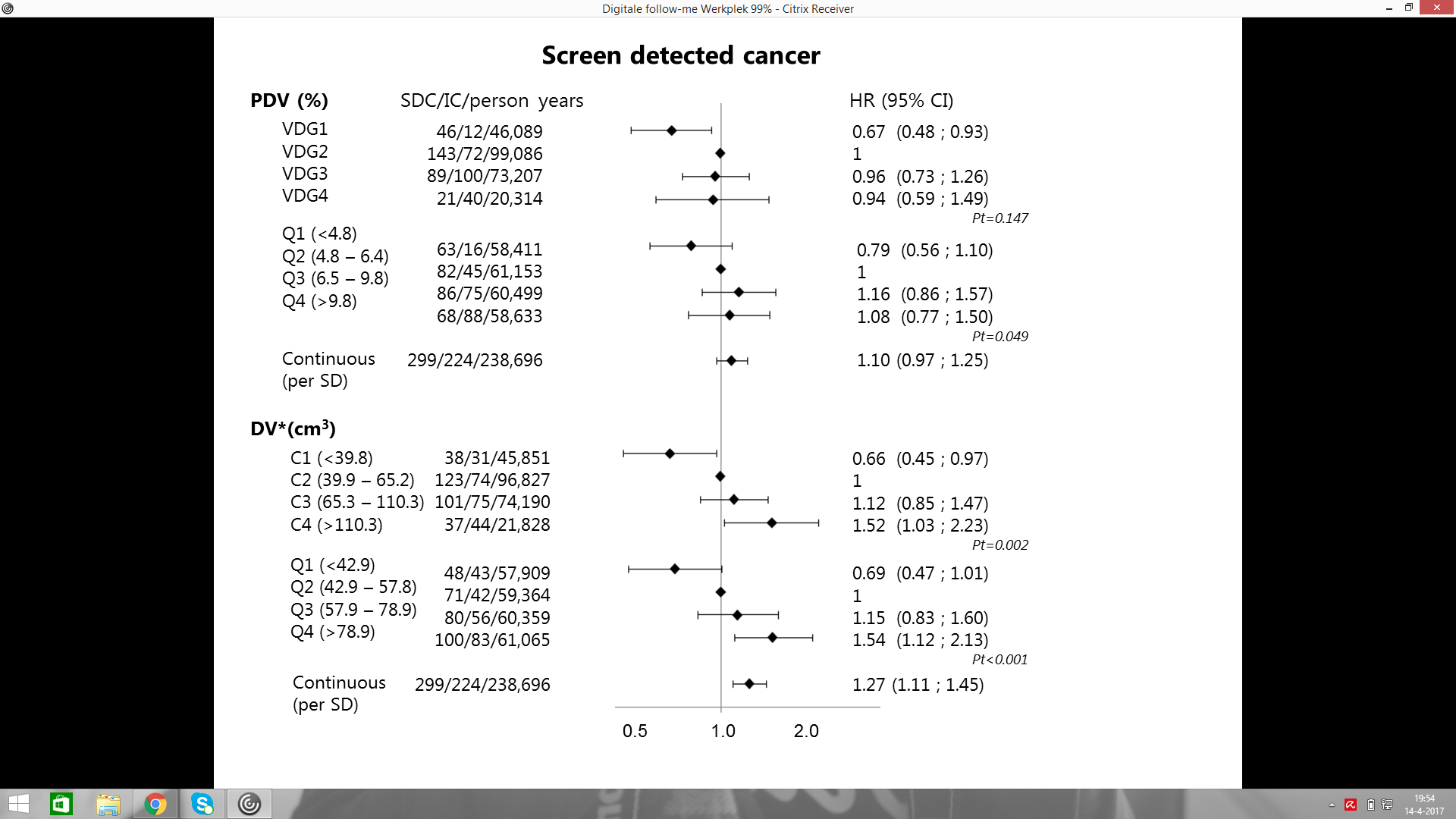

Supplement: Supplementary file 2 — Mammographic measures and screen-detected, and interval breast cancer risk (second category used as reference). Lunn and McNeil method was used for competing risk analysis. Pt p trend: this was determined by adding the categorical measures as a continuous measure into the model, PDV percentage dense volume, DV dense volume, Per SD per standard deviation, SDC screen-detected cancer, IC interval cancer. *Absolute dense volume measures are adjusted for nondense (breast fat) volume. (DOCX 890 kb) [file 13058_2017_859_MOESM2_ESM.docx]
