# Supplementary material for: The effect of volumetric breast density on the risk of screen-detected and interval breast cancers: a cohort study
Source: Breast Cancer Res. 2017 Jun 5;19:67. doi: 10.1186/s13058-017-0859-9 (PMC5460501; doi:10.1186/s13058-017-0859-9)
Supplement: Supplementary file 3 — Overview of associations between volumetric breast density measures (measured with Volpara) and breast cancer risk. Overview of studies that also determined the association between Volpara density grade categories and breast cancer risk. (DOCX 23 kb) [file 13058_2017_859_MOESM3_ESM.docx]

**Additional file 3: Table S1 – Overview of associations between volumetric breast density measures (measured with Volpara) and breast cancer risk.**

|  | **Study design &  sample size** (cases/ non cases) | **VDG** OR/HR (95% CI) | **PDV** OR/HR (95% CI)  (per SD increase) | **DV** OR/HR (95% CI)  (per SD increase) | Adjusted for: |
| --- | --- | --- | --- | --- | --- |
| Wanders et al.  (current study) | Prospective cohort  (523/52,814) | VDG 4 vs VDG 1:  3.14 (2.17 ; 4.55)  VDG 4 vs VDG2: 1.76 (1.32 ; 2.37) | 1.36 (1.25 ; 1.48) | 1.45 (1.32 ; 1.60) | Age, DV additionally adjusted for NDV |
| Park et al. (2014)[37] | Case-control (677/1,307) | VDG 4 vs VDG 1+2:  3.07 (1.89 ; 4.99) | NA | NA | Age, BMI, parity, postmenopausal  HRT use (postmenpausal women  only) |
| Eng et al. (2014)[15] | Case-control (414/685) | NA | 1.75 (1.44 ; 2.11) | 1.38 (1.18 ; 1.61) | Age, BMI, menopausal status, parity |
| Brand et al. (2014)[16] | Prospective cohort (206/40,896) | NA | 1.46 (1.22 ; 1.76) | 1.30 (1.12 ; 1.51) | Age, BMI, menopausal status, parity,  age at first birth, HRT status, benign breast disease, family history of BC |
| Brandt et al. (2015)[17] | Case-control  (1,911/4,170) | VDG 4 vs VDG 2:  1.82 (1.49 ; 2.21) | 1.47 (1.36 ; 1.60) | 1.31 (1.23 ; 1.40) | Age, BMI |
| Jeffers et al. (2016)[18] | Case-control (125/274) | VDG 4 vs VDG 2: 2.05 (0.90 ; 4.64) | 1.54 (1.12 ; 2.10) | 1.41 (1.11 ; 1,80) | BMI, menopausal status, parity  (matched on age and race) |

VDG: Volpara Density Grades, PDV: Percent Dense Volume, DV: Dense Volume, NDV: Nondense Volume, SD: Standard Deviation, BMI: Body Mass Index, HRT: Hormone Replacement Therapy, BC: Breast Cancer.
